# First use of pulsed field ablation in a patient with a deep brain stimulator: a case report

**DOI:** 10.1093/ehjcr/ytag560

**Published:** 2026-07-30

**Authors:** Alexandra Marx, David Schaack, K R Julian Chun, Boris Schmidt, Lukas Urbanek

**Affiliations:** Cardioangiologisches Centrum Bethanien, Agaplesion Markus-Krankenhaus, Wilhelm-Epstein- Str. 4, Frankfurt/M 60431, Germany; Cardioangiologisches Centrum Bethanien, Agaplesion Markus-Krankenhaus, Wilhelm-Epstein- Str. 4, Frankfurt/M 60431, Germany; Cardioangiologisches Centrum Bethanien, Agaplesion Markus-Krankenhaus, Wilhelm-Epstein- Str. 4, Frankfurt/M 60431, Germany; Department of Rhythmology, University Hospital Schleswig-Holstein, Campus Lübeck, University of Lübeck, Ratzeburger Allee 160, 23538 Lübeck, Germany; Cardioangiologisches Centrum Bethanien, Agaplesion Markus-Krankenhaus, Wilhelm-Epstein- Str. 4, Frankfurt/M 60431, Germany; Department of Cardiology and Angiology, University Hospital Frankfurt, Theodor-Stern-Kai 7, 60596 Frankfurt am Main, Germany; Cardioangiologisches Centrum Bethanien, Agaplesion Markus-Krankenhaus, Wilhelm-Epstein- Str. 4, Frankfurt/M 60431, Germany; Department of Cardiology, University Medical Center Mainz, Johannes Gutenberg University Mainz, Langenbeckstraße 1, 55131 Mainz, Germany

**Keywords:** Pulsed field ablation, Atrial fibrillation, Deep brain stimulator, Neurostimulation device, Case report

## Abstract

**Background:**

Pulmonary vein isolation for atrial fibrillation (AF) in patients with implanted neurostimulation devices is challenging, as established energy sources, such as radiofrequency (RF) ablation, are generally not recommended in these patients due to the risk of device interference. Pulsed field ablation (PFA) for AF offers relative myocardial selectivity and a potentially improved safety profile. However, data on its use in patients with implanted neurostimulation devices are lacking.

**Case summary:**

A 66-year-old woman with paroxysmal AF was referred to our centre for catheter ablation. Years earlier, a deep brain stimulator (DBS) had been implanted to control severe fasciculations. As RF ablation is not recommended in such patients and given the patient’s multiple comorbidities, a single-shot ablation strategy was chosen. After selective pulmonary vein angiography revealed a large left common pulmonary vein (LCPV), PFA using a pentaspline catheter was selected, as this approach allows effective treatment of a LCPV with short procedure times and a favourable safety profile. The DBS was deactivated prior to the procedure and ablation was performed without complications. After the procedure, the DBS was reactivated and showed no evidence of device malfunction.

**Discussion:**

This is the first reported case that demonstrates the feasibility of PFA in a patient with an implanted DBS. Temporary deactivation of the neurostimulation device and careful multidisciplinary planning allowed successful ablation without device dysfunction or interference. These findings suggest that, with appropriate precautions, PFA may represent a viable ablation strategy in selected patients with implanted neurostimulation systems.

Learning points

- Pulsed field ablation can be considered in selected patients with an implanted deep brain stimulator after careful multidisciplinary evaluation.
- Temporary deactivation of the deep brain stimulator before ablation with post-procedural reactivation and functional testing is an important procedural precaution.
- Pulsed field ablation using a pentaspline catheter was performed without observable interference with a pectorally implanted neurostimulation device.

## Introduction

According to the 2024 ESC guidelines for the management of atrial fibrillation (AF), catheter ablation is the first-line therapy for symptomatic paroxysmal AF to reduce symptoms, recurrence, and progression of AF, and the cornerstone of catheter ablation for AF is pulmonary vein isolation (PVI).^1^ Notably, current ESC guidelines do not provide specific recommendations regarding catheter ablation in patients with implanted deep brain stimulators (DBSs), and evidence regarding the use of different ablation energy modalities in this population remains lacking.

While thermal energy sources, such as radiofrequency (RF) ablation, or single-shot technologies, such as the cryoballoon (CB), are widely used in the general population, their application in patients with implanted neurostimulation devices, including DBSs, is limited.

Radiofrequency ablation is generally not recommended in patients with implanted DBSs because of the potential risk of electromagnetic interference, which may result in inappropriate neurostimulation, device malfunction, or unintended tissue heating along the lead system.^2^ These concerns are also reflected in manufacturer instructions for use, which caution against the application of RF energy in patients with implanted neurostimulation devices (Boston Scientific, Model DB-1216 Vercise Genus R16). Alternatively, there are isolated reports describing the safe use of CB ablation in patients with DBSs.^3^ However, clinical experience with cryothermal ablation in this specific patient population remains limited, largely restricted to single case reports, and systematic data on procedural safety and device interaction are lacking.

Pulsed field ablation (PFA) is a non-thermal ablation modality based on irreversible electroporation, which creates myocardial-selective lesions while largely sparing adjacent structures. Early clinical studies have demonstrated high acute success rates, short procedure durations, and a favourable safety profile for PVI.^4^

Nevertheless, PFA is not entirely free of extracardiac effects, and complications, such as coronary artery spasm, have been reported.^5^ With the increasing prevalence of AF, a growing number of patients undergoing catheter ablation also carry cardiac implantable electronic devices (CIEDs), including pacemakers or implantable cardioverter-defibrillators. Initial reports suggest that PFA-based PVI can be performed safely in patients with CIEDs when systematic peri-procedural device interrogation and management are applied.^6^ However, potential electromagnetic interference with CIEDs have been described, particularly when ablation is performed in proximity to critical structures.^6,7^ Data on the interaction between PFA and deeply implanted neurostimulation systems, such as DBSs, are lacking.

## Summary figure

**Figure:**
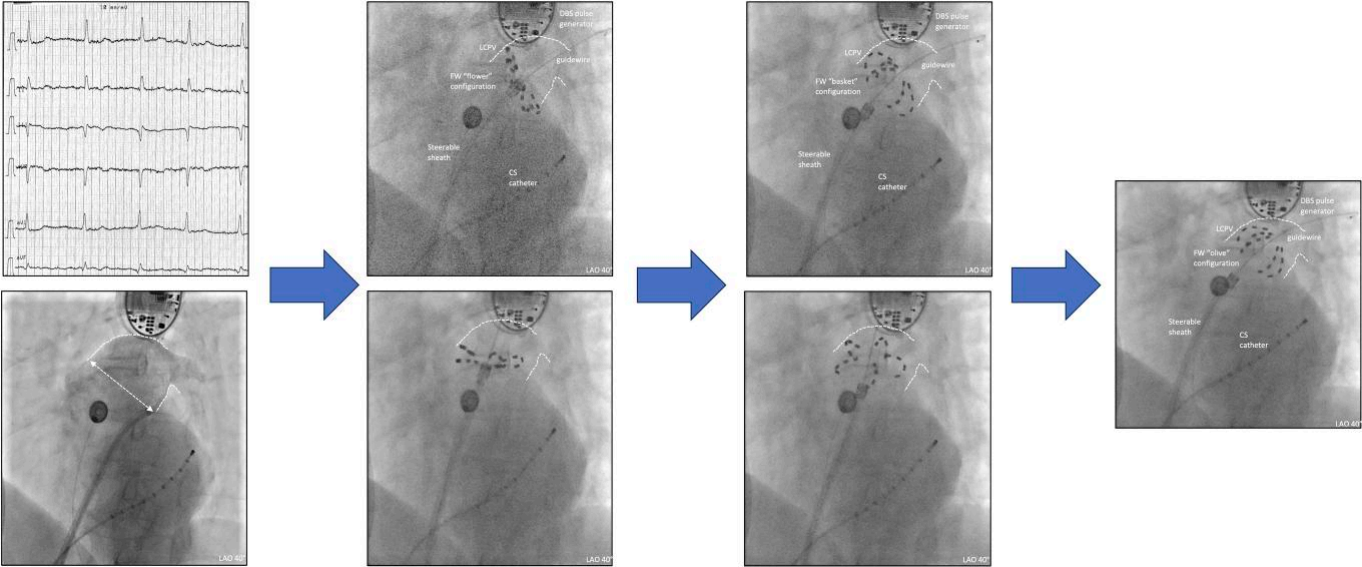


## Case presentation

A 66-year-old female patient with a history of muscular dystonia and severe fasciculations was referred for catheter ablation due to highly symptomatic paroxysmal AF. The patient was considered frail due to multiple comorbidities, including a history of gastrointestinal bleeding and unexplained weight loss, together with reduced clinical reserve and relevant functional impairment. Moreover, deactivation of deep brain stimulation was associated with pronounced fasciculations, severe gait disturbance, impaired mobility, and substantial limitations in activities of daily living.

Several years earlier, a DBS had been implanted to control her neurological symptoms, with the pulse generator located subcutaneously in the pectoral position.

The two-lead DBS system stimulated successfully the globus pallidus internus in each hemisphere in a continuous way with high-frequency impulses. The case was discussed in a multidisciplinary setting with a cardiologist and her neurologists.

**Figure 1:**
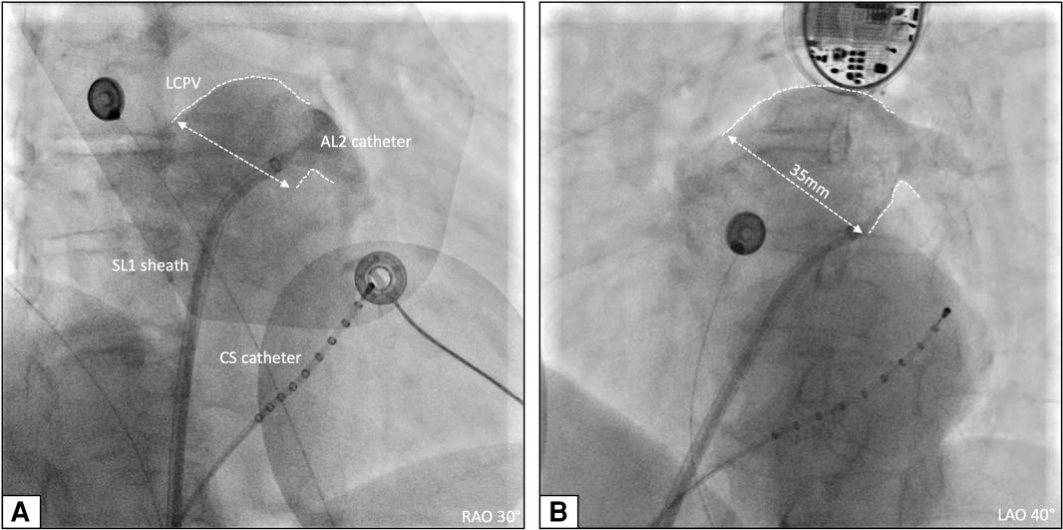
(*A*) Selective pulmonary vein angiography of the left common pulmonary vein in a 30° right anterior oblique projection. (*B*) Selective pulmonary vein angiography of the left common pulmonary vein in a 40° left anterior oblique projection. AL2, Amplatz Left 2; CS, coronary sinus; LAO, left anterior oblique; LCPV, left common pulmonary vein; RAO, right anterior oblique.

**Figure 2:**
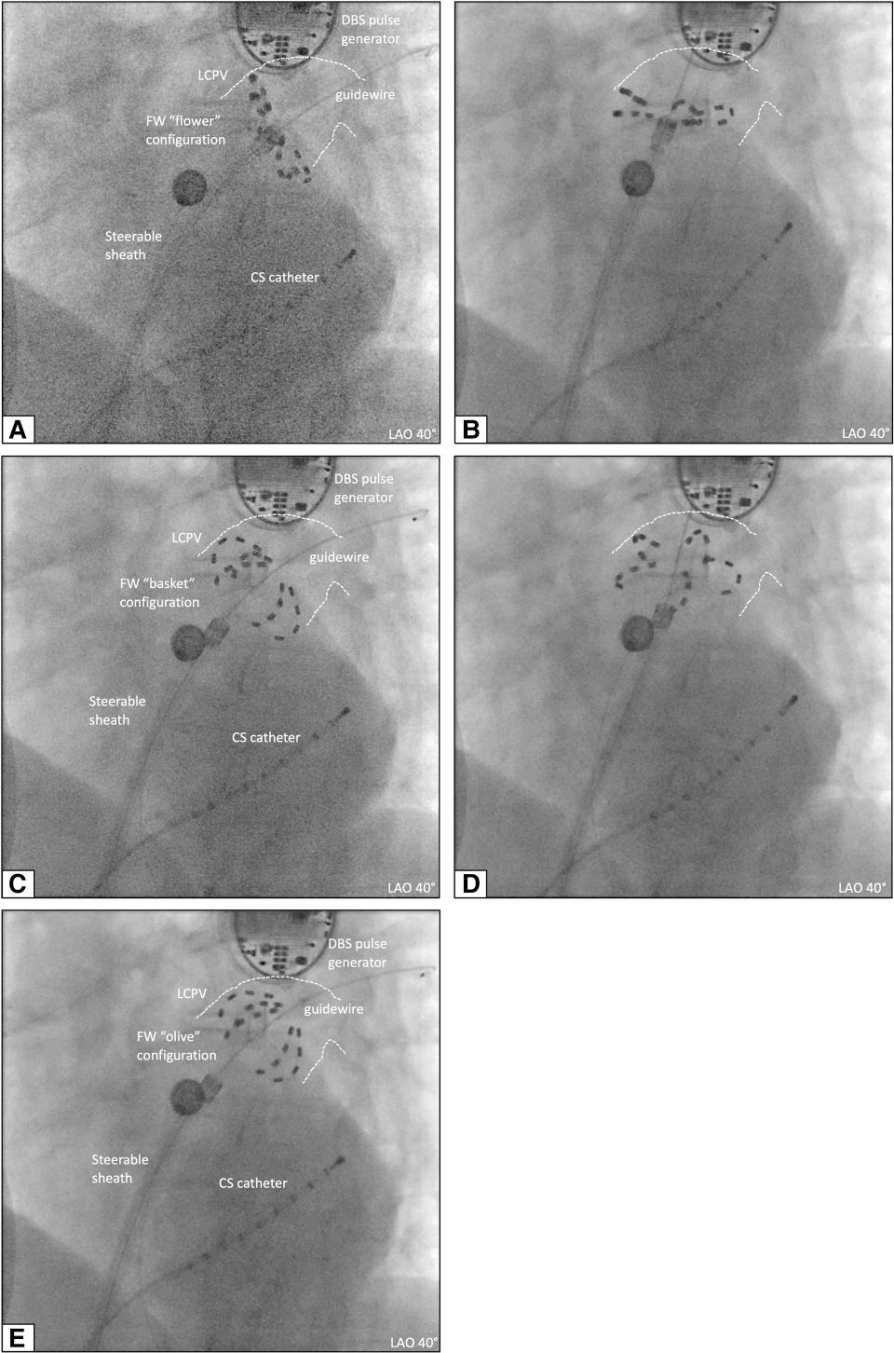
(*A*) FARAWAVE catheter (31 mm) in a flower configuration at the ostium of the left common pulmonary vein. The guidewire is positioned in an inferior branch of the left common pulmonary vein, providing good contact of the splines, particularly with the inferior aspect of the left common pulmonary vein. The close proximity of the ablation catheter to the deep brain stimulation generator is clearly visible. (*B*) FARAWAVE catheter (31 mm) in a flower configuration at the ostium of the left common pulmonary vein. The guidewire is positioned in a superior branch of the left common pulmonary vein, providing good contact of the splines, particularly with the superior aspect of the left common pulmonary vein. (*C*) FARAWAVE catheter (31 mm) in a basket configuration at the ostium of the left common pulmonary vein. The guidewire is positioned in an inferior branch of the left common pulmonary vein. (*D*) FARAWAVE catheter (31 mm) in a basket configuration at the ostium of the left common pulmonary vein. The guidewire is positioned in a superior branch of the left common pulmonary vein. (*E*) FARAWAVE catheter (31 mm) in an olive configuration with a slight protrusion of the catheter into the vein. The guidewire is positioned in an inferior branch of the left common pulmonary vein. CS, coronary sinus; DBS, deep brain stimulator; LAO, left anterior oblique; LCPV, left common pulmonary vein; FW, FARAWAVE

As RF harbours the risk of electromagnetic interference and tissue heating along the lead system, RF was not considered for AF ablation in this patient.^2^ Alternatively, CB ablation was discussed, as there are isolated reports describing its safe use in patients with implanted DBSs.^3^ However, given the patient’s multiple comorbidities and reduced general condition, PFA was also considered because of its short procedure times and favourable safety profile, as well as its ability to effectively treat pulmonary vein anatomies that are not amenable to CB ablation, such as an LCPV. It was ultimately decided to initiate the procedure and to reassess intra-procedurally, taking into account the patient’s tolerance of sedation and the pulmonary vein anatomy as assessed by selective pulmonary vein angiography, before making a final decision between CB ablation and PFA. As a precautionary measure, the DBS was deactivated immediately prior to the ablation procedure. The procedure was performed under deep sedation using propofol, midazolam, and fentanyl. After pulmonary vein angiography (Figure 1) revealed a large LCPV not suitable for CB ablation, the decision was made to perform PFA using the pentaspline catheter and a 31 mm FARAWAVE (FW) catheter with a sequential ablation approach (Figure 2), despite a measured LCPV diameter of ∼35 mm. The 35 mm device was considered less suitable, as its nominal diameter of ∼35 mm is primarily achieved in the flower configuration, posing a risk that in alternative configurations the catheter could advance too deeply into the vein; therefore, a sequential ablation approach would likely have been required with both the 31 mm and the 35 mm devices. Given the relatively small left atrial size, the use of a 35 mm device in a sequential strategy carried a potential risk of excessive atrial ablation, particularly with close lesion proximity between the left- and right-sided pulmonary veins along the posterior wall and roof. This could theoretically promote the formation of a roof-related slow-conduction corridor and thereby increase the risk of organized atrial tachycardia. Consequently, a sequential ablation strategy using the 31 mm device was adopted. The LCPV was treated in a segmental manner with 20 applications, targeting the inferior and superior portions separately (Figure 2) to ensure adequate lesion coverage while maintaining stable catheter positioning. Pulsed field ablation applications were delivered using the pentaspline catheter in basket and flower configurations, with four applications per configuration and catheter rotation of ∼36° after two applications to optimize lesion overlap. In addition, two applications in the so-called olive configuration were delivered deeper within each pulmonary vein to target the pulmonary vein sleeves. The procedure was limited to PVI, without additional substrate modification or extra-pulmonary vein lesions, given the patient’s paroxysmal AF and non-dilated left atrium. In total, 40 PFA applications were delivered across all pulmonary veins.

The procedure time was 40 min, and throughout the procedure, no abnormal signals, device interference, or unexpected electrical phenomena were observed. After completion of the ablation, the DBS was reactivated, and comprehensive device interrogation confirmed normal function without parameter changes or malfunction. Beyond the immediate post-procedural DBS assessment, follow-up interrogations performed by the Department of Neurosurgery confirmed continued proper device function without evidence of device impairment or functional restrictions, most recently in early 2026.

## Discussion

This case represents the first reported use of PFA in a patient with an implanted DBS in whom RF was not possible due to the risk of electromagnetic interference and tissue heating along the lead system.^2^ While theoretical concerns existed regarding electromagnetic interference due to high-voltage pulse delivery, no device interaction, generator reset, or parameter change was observed in our patient. Temporary deactivation of the DBS before energy delivery and systematic post-procedural interrogation proved to be a pragmatic and safe strategy.

Deep brain stimulation systems consist of intracranial electrode leads implanted in defined deep brain targets, such as the globus pallidus internus in the present patient. These leads are connected via subcutaneous extension cables, which are tunnelled through the neck to an implantable pulse generator typically located in the pectoral region. Although the overall architecture of a DBS system resembles that of a cardiac pacemaker, with implanted leads connected to a pulse generator via extension cables, DBS delivers continuous programmable high-frequency neuromodulation rather than sensing-dependent, event-triggered cardiac pacing.

Although several reports have described PFA in patients with cardiac implantable electronic devices as feasible and uneventful, emerging data indicate that device-related interactions may occur. Pulsed field ablation may induce electromagnetic interference within the frequency range between the high- and low-pass filters of CIEDs, potentially leading to oversensing.^8,9^ The occurrence and extent of such interactions likely depend on several factors, including application site, pulse duration, polarity, waveform characteristics, catheter-tissue contact, and the total energy delivered per application.^10^ Importantly, despite these observations, clinically relevant device malfunction remains rare, and most reported events are transient and reversible.^7^ These observations support careful peri-procedural device assessment and monitoring when PFA is performed in patients with implanted cardiac devices.

A second aspect of this case was the presence of a large left common pulmonary vein (LCPV). Left common pulmonary vein anatomy can limit the effectiveness of certain single-shot devices, particularly CB systems, where complete occlusion and homogeneous lesion formation may be difficult to achieve.^11^ In this context, pentaspline PFA represented a particularly suitable single-shot alternative.

In this case, the flexible catheter design allowed sequential ablation of the individual LCPV branches in different configurations (flower, basket, olive), ensuring circumferential ostial coverage while maintaining stable catheter positioning.^12^ By targeting the superior and inferior aspects of the common trunk separately, effective isolation was achieved without the need for extensive atrial ablation. This branch-oriented approach may be especially advantageous in large common ostia, where tailored lesion deployment is required. We deliberately selected the 31 mm pentaspline catheter. Despite the diameter of the LCPV measuring 35 mm, we assumed that the 31 mm device would provide a more homogeneous electric field distribution at the ostial level and allow effective energy delivery with stable spline contact at both the superior and inferior aspects of the common vein. In contrast, the 35 mm catheter, although larger in nominal diameter, achieves its maximal expansion primarily in the flower configuration and may not ensure uniform ostial coverage in all configurations in a non-sequential approach. Moreover, its use could have required deeper advancement into the vein or could have resulted in an excessive atrial ablation with a sequential strategy, which could promote the formation of a roof-related slow-conduction corridor and thereby increase the risk of organized atrial tachycardia without necessarily improving ostial coverage.^13^ Therefore, the 31 mm device was considered the more controlled and anatomically appropriate option for achieving effective branch-based LCPV isolation.

Beyond anatomical considerations, PFA offers additional advantages in clinically vulnerable patients. Large multicentre data have demonstrated a favourable safety profile with low rates of collateral injury.^10,14^ The non-thermal mechanism of action reduces the risk of oesophageal damage, phrenic nerve palsy, or pulmonary vein stenosis, which is particularly relevant in frail patients with multiple comorbidities. Moreover, the short procedure time in our case (40 min) reflects one of the practical strengths of single-shot PFA. Reduced procedural duration may lower sedation requirements and contribute to overall procedural safety in patients with limited physiological reserve.

In summary, this case illustrates that pentaspline PFA enables effective branch-based isolation of a large LCPV and may represent an attractive single-shot strategy when other balloon-based systems are anatomically suboptimal and RF ablation is relatively contraindicated.

## Conclusion

Pulsed field ablation using a pentaspline single-shot system appears to be feasible and safe in a patient with an implanted DBS when appropriate peri-procedural precautions are applied. This case provides important preliminary evidence supporting the use of PFA in patients with neurostimulation devices and highlights the need for further systematic evaluation.

## Lead author biography


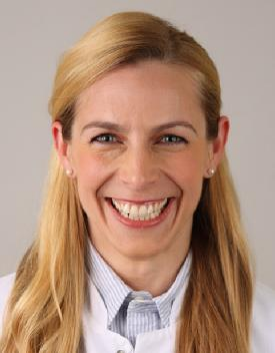


Alexandra Marx is a cardiologist and researcher at Cardiologisches Centrum Bethanien in Frankfurt, Germany. Her clinical and scientific focus lies on catheter ablation of AF.

## Data Availability

The data underlying this article are available from the corresponding author upon reasonable request.
